# The British Hospitals' Association

**Published:** 1911-06-10

**Authors:** Conrad W. Thies

**Affiliations:** Hon. Treasurer. Royal Free Hospital, Gray's Inn Road, W.C.


### THE BRITISH HOSPITALS' ASSOCIATION.

*To the Editor of* THE HOSPITAL.

SIR,—The meeting which was held at St. George's Hospital on the 2nd inst. to consider the probable effects of the National Insurance Bill upon the voluntary hospitals was convened by the Council of the British Hospitals Association. As many of those present were not informed as to the objects of the Association, I may state that it was formed last year for the purpose of affording facilities for the consideration and discussion of matters connected with hospital management and to take measures to further the decisions arrived at. The Association also undertook to arrange for the reading, discussion, and publication of papers, and for the holding of an annual conference for the consideration of matters relating to hospital administration generally. The first annual conference was held in Glasgow in September 1910, and arrangements are now being made for holding the second conference in Manchester on September 28 and 29 next. All persons who are engaged in the active administration of hospitals, either as trustees, members of committee, or as executive officers, are eligible for membership upon payment of an annual subscription of half a guinea. As the Association has to bear very considerable expense in organising meetings and the issue of papers, etc., it is essential that the membership should be very greatly increased if its usefulness is to be extended. I shall be glad to receive applications for membership.—Yours faithfully,

CONRAD W. THIES,
Hon. Treasurer.

Royal Free Hospital,
Gray's Inn Road, W.C.,
June 7.

